# Particulate matter (PM_10_) prediction based on multiple linear regression: a case study in Chiang Rai Province, Thailand

**DOI:** 10.1186/s12889-021-12217-2

**Published:** 2021-11-24

**Authors:** Wissanupong Kliengchuay, Rachodbun Srimanus, Wechapraan Srimanus, Sarima Niampradit, Nopadol Preecha, Rachaneekorn Mingkhwan, Suwalee Worakhunpiset, Yanin Limpanont, Kamontat Moonsri, Kraichat Tantrakarnapa

**Affiliations:** 1 Department of Social and Environmental Medicine, Faculty of Tropical Medicine, Mahidol University, Bangkok, Thailand; 2 Environment, Health & Social Impact Unit, Department of Social and Environmental Medicine, Faculty of Tropical Medicine, Mahidol University, Bangkok, Thailand; 3 School of Medicine, St. George’s University, Saint George’s, West Indies Grenada; 4 School of Public Health, Walailak University, Nakhorn Sri Thammarat, Thailand; 5 The Graduate School of Environmental Development Administration, Bangkok, Thailand

## Abstract

**Background:**

The northern regions of Thailand have been facing haze episodes and transboundary air pollution every year in which particulate matter, particularly PM_10_, accumulates in the air, detrimentally affecting human health. Chiang Rai province is one of the country’s most popular tourist destinations as well as an important economic hub. This study aims to develop and compare the best-fitted model for PM_10_ prediction for different seasons using meteorological factors.

**Method:**

The air pollution and weather data acquired from the Pollution Control Department (PCD) spanned from the years 2011 until 2018 at two stations on an hourly basis. Four different stepwise Multiple Linear Regression (MLR) models for predicting the PM_10_ concentration were then developed, namely annual, summer, rainy, and winter seasons.

**Results:**

The maximum daily PM_10_ concentration was observed in the summer season for both stations. The minimum daily concentration was detected in the rainy season. The seasonal variation of PM_10_ was significantly different for both stations. CO was moderately related to PM_10_ in the summer season. The PM_10_ summer model was the best MLR model to predict PM_10_ during haze episodes. In both stations, it revealed an R^2^ of 0.73 and 0.61 in stations 65 and 71, respectively. Relative humidity and atmospheric pressure display negative relationships, although temperature is positively correlated with PM_10_ concentrations in summer and rainy seasons. Whereas pressure plays a positive relationship with PM_10_ in the winter season.

**Conclusions:**

In conclusion, the MLR models are effective at estimating PM_10_ concentrations at the local level for each seasonal. The annual MLR model at both stations indicates a good prediction with an R^2^ of 0.61 and 0.52 for stations 65 and 73, respectively.

**Supplementary Information:**

The online version contains supplementary material available at 10.1186/s12889-021-12217-2.

## Background

Atmospheric pollution distributions are recognized as complicated challenges all over the world, especially in developing countries [1]. Many researchers ascribe the temporal pattern of air pollutants to the combined effect of many factors, each one with its seasonality: atmospheric and hydrological processes, human activities, long-range transport, natural emissions, and extreme events [2]. The pollution in Southeast Asia is due to both natural factors and human activity. The anthropogenic sources are transportation, industrial processes, household activities, and agricultural burning. Moreover, pollutants are released naturally from forest fires. Many common characteristics of ASEAN countries will be tropical climatic conditions, which can result in extreme temperatures, rainfall, and high relative humidity. In addition, biomass burning is a major regional source of particulate matter in the atmosphere, most notably during the dry seasons [3]. These features introduce a large variability of haze characteristics distributed over this region. It was almost a decade ago that these regions started experiencing air quality problems that the haze episodes brought annually in the upper north of Thailand [4, 5]. Almost all eight provinces in the upper north of Thailand are mountainous ranges and valleys. Identifying the transboundary of haze in tropical mountain cities will contribute to a growing body of knowledge currently being developed in different parts of world [6]. The particulate matter (PM) is an important pollutant present in the atmosphere that can penetrate the respiratory system and is a health hazard. High concentrations of particulate matter have caused disturbances to the environment, such as degraded atmospheric visibility, and to human health, such as acute or chronic respiratory diseases [7–9].

Thailand is one of many countries in this region that have had environmental concerns. During the dry season every year, the north of Thailand experiences haze episodes. PM_10_ is one of the key factors for government monitoring and surveillance by the Pollution Control Department (PCD), Ministry of Natural Resources and Environment, Thailand. Haze is determined when average daily concentrations exceed 120 μg/ m^3^ (National Ambient Air Quality Standard) [10]. Chiang Rai is a popular tourist destination and the northernmost province of Thailand, bordered by the Shan state of Myanmar and the Bokeo province of Laos. Chiang Rai has a total area of 11,678.37 km2 and a population of 1.28 million. This province is suffering from various air pollution factors, such as haze transboundary, biomass burning, and forest fires, [11]. From March 2014 to 2016, researchers studied the PM_10_ measurement station in Chiang Rai province and discovered that 51, 28 and 21% of the hotspots in Myanmar, Lao PDR, and Thailand, respectively, primarily moved across the province’s south-western border. Haze has emerged every year during the transition between the cold and dry seasons. The haze episode caused not only an air pollution problem, it also affected the socio-economics in this province. Tourist activities and related services were cancelled due to the haze problem. There might be benefits for all related sectors in preparing for the unpredictable event. This study aims to support the local organization to forecast the haze episode by using the available monitored data. The overview of air pollution in this study focuses on the investigation of the correlation between air pollutants (PM_10_) and meteorological parameters. Statistical studies using meteorological data and air pollution monitoring data have confirmed that meteorological conditions affect atmospheric pollution in numerous ways [1]. However, the most important role of meteorology is the effect on the dispersion, transformation, and removal of atmospheric pollutants from the atmosphere and finally affects the spatial-temporal characteristics and pollution levels of atmospheric pollutants. Some researchers reported that the meteorological factors influencing PM_10_, such as wind direction and speed, pressure, relative humidity, etc. This study therefore investigated their relationships in different scenarios, such as throughout the year and seasonal variation. The weather in different seasons might have influenced the PM_10_ only in some seasons. This study focuses on the following: (1) Investigating the temporal variations of PM_10_ in Chiang Rai, Thailand, between 2011 and 2018; and (2) Examining the effect of meteorological and air pollution factors on the seasonal variation of PM_10_ concentration distribution. (3) the establishment of MLR models for the three different seasons in Chiang Rai province. The outcomes of this study give insight into the sources of pollutants in Chiang Rai, and how pollutant behavior is influenced by concentrations and factors of interrelationships in pollutant behavior. The results can be used for information distribution to local communities and people for their response and preparation. In addition, our findings will be beneficial in supporting the sustainable development goals (SDGs), particularly targets 13 (Climate Action), 3 (Good Health and Well Being), 12 (Sustainable Consumption and Production), and 17 (Partnership). Referring to target 13, climate action might be the drive or pressure to reduce the use of fossil fuels and GHG (Green House Gas) emissions reduction. As stated in target 12, air pollution and GHG emissions are linked to fossil fuel consumption and human activities. Target 3 is the consequence of human activities. Good health and wellbeing are directly linked to the environment, such as air quality and socio-economic status. In order to achieve the goal for each target, collaboration among various organizations in both national and international networks is needed to strengthen it.

## Methods

### Study area and data collection

**Fig. 1:**
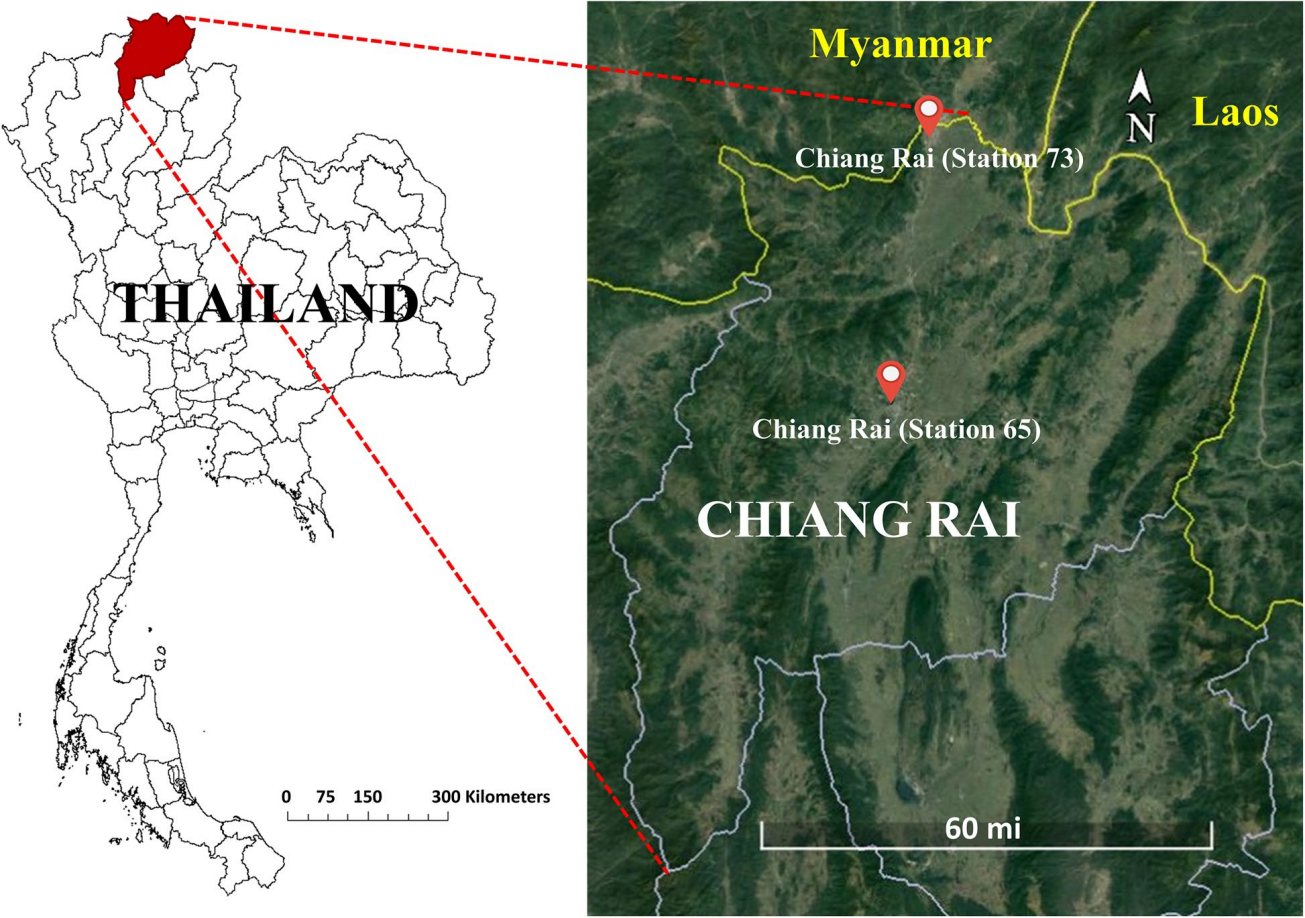
Chiang Rai air quality monitoring stations (PCD station 65 and 73)

Transboundary haze events are caused by large-scale biomass combustion in the northern parts of Thailand. The haze events usually occur during the months of mid-February to mid-May (dry season) every year. Figure 1 shows the location of the affected area, where air pollution data was obtained from the Pollution Control Department (PCD), Thailand observation station. In particular, the majority of PM data available has been collected using the Beta ray absorption or Beta-gauge attenuator, and the Tapered Element Oscillating Microbalance (TEOM) techniques have been used, including air quality monitoring stations in Chiang Rai province. Daily PM_10_ concentration data were collected at two stations for 7 years, from January 1, 2011, to December 31, 2018 (station 65) and from April 1, 2011, to December 31, 2018 (station 73).

### Statistical and temporal analysis

This is an annual analysis of daily PM_10_ from 2011 to 2018 at the Chiang Rai station (65 and 73). The data was tabulated using Microsoft Excel Spreadsheet® and analysis of the data were carried out using statistical software, R-studio open air package. The Bonferroni correction multiple comparison test was used to estimate differences between mean concentrations of PM_10_ among seasonal periods across the year at 5%, and Spearman’s rank correlation coefficient aimed to determine the interaction between PM_10_ and meteorological factors.

The MLR model is essential in determining how the meteorological factors affect air pollutant concentrations. Thus, the PM_10_ concentrations can be treated as a response to the meteorological variables as predictors. The model is itemized in Equation [12].

1 $$y={b}_0+\sum {i}^n={{}_1b}_i{x}_i+\varepsilon$$

where, *y* is the dependent variable, *b*_0_ is the regression intercept (constant term), *b*_*i*_ is the regression coefficient (independent variables), *x*_*i*_ is the explanatory variable, ε is the stochastic error associated with the regression. For analysis, the multicollinearity is defined as the variance inflation factor (VIF) to calculate for meteorological factors in these models. The multicollinearity analysis is used for independent variables. Our independent variables were both air quality data and meteorological data. Therefore, it is assumed that multicollinearity between selected predictors is not present [13, 14].

### Trajectory models

The HYSPLIT (hybrid single particle Lagrangian integrated trajectory) model [15] has been applied in most of the studies. The airmasses are responsible for the export and import of pollutants deposited in the country and neighboring areas [16–18]. Formalized paraphrase. The focus of this study was on the back trajectories of air parcels detected at 2 air quality monitoring stations in Chiang Rai Province. The direction analysis of air mass movement in reverse, which selected the date of the highest PM_10_ at the top of each year, considered a period of 24 h.

## Results and discussion

### Descriptive statistics

The characteristics of PM_10_ data from 2011 to 2018 in Chiang Rai province are summarized in Table 1; The daily PM_10_ concentration is greater than the national ambient air quality standard (NAAQS) of 120 μg/m^3^. The maximum 24-h concentrations of PM_10_ were 371.1 and 129.6 μg/m^3^ at stations 65 and 73, respectively. The annual average concentration was 41.9 at station 65, which was slightly higher than at station 73 (37.4 μg/m^3^). However, the maximum concentration can be detected at any time of the day.

**Table 1:** Descriptive statistics of daily PM_10_ concentrations during 2011 to 2018

Variable PM_10_	N	Minimum (μg/m^3^)	Maximum (μg/m^3^)	Mean (μg/m^3^)	Std. deviation
**Station 65**					
Annual	2922	1.6	371.1	41.9	35.5
Summer	714	8.5	371.1	78.6	53.2
Rainy	1224	1.6	80.6	23.2	10.2
Winter	984	6.8	130.3	40.4	17.3
**Station 73**					
Annual	2741	3.00	129.6	37.4	22.8
Summer	625	12.2	129.6	56.1	25.8
Rainy	1178	3.0	80.2	27.1	14.5
Winter	938	3.0	80.2	27.6	15.5

**Fig. 2:**
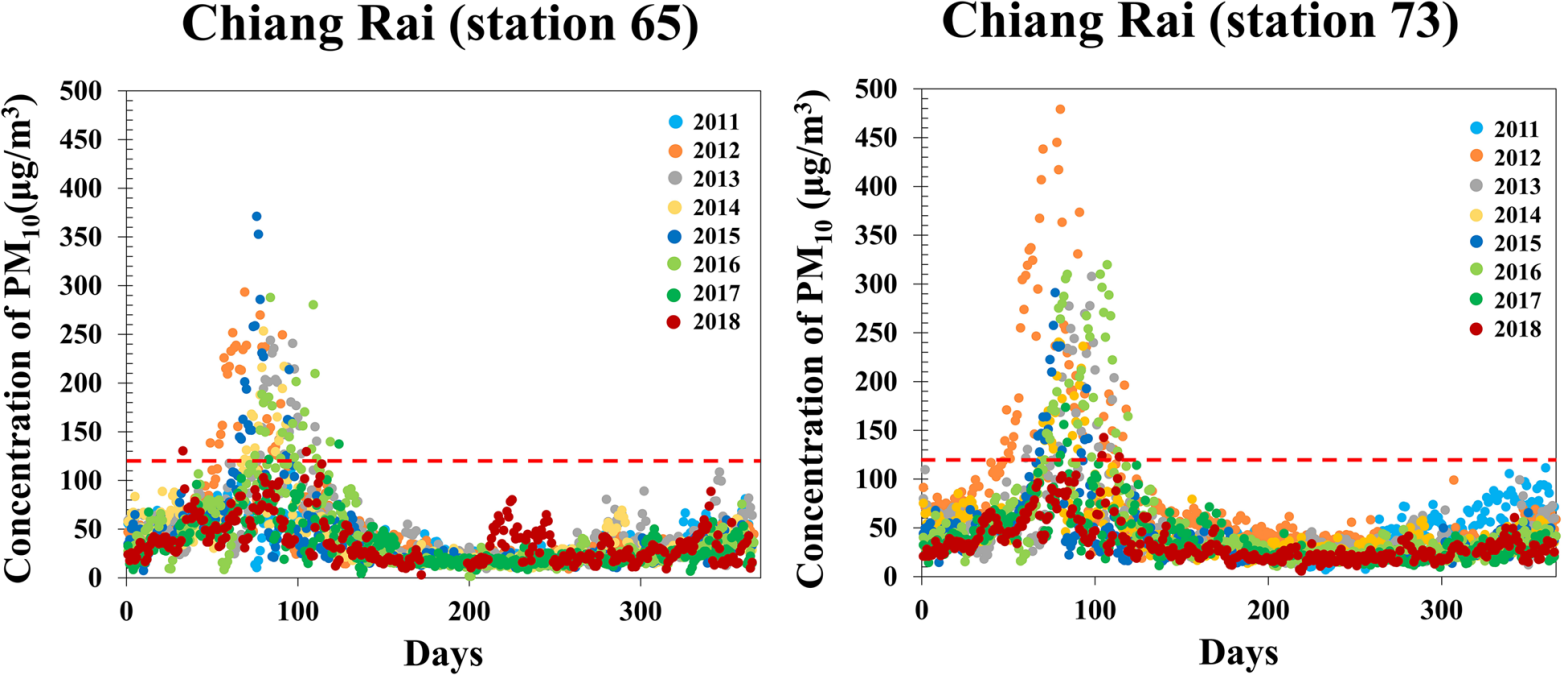
Seasonal variable of PM_10_ concentration plot from 2011to 2018 in Chiang Rai

Figure 2 shows that the daily average concentration of PM_10_ presents a similar pattern during the year 2011 to 2018. This figure shows the behavior of PM_10_ concentrations at different times. The concentration of PM_10_ seems to have a similar trend from the start of the year to the end of the year, whereas maximum (summer) and minimum (rainy) concentrations occur at different times. While considering seasonal variations of PM_10_ was higher during the summer compared to another season. Similarly, both station concentrations of PM_10_ were higher in 2012, 2013, 2014, 2016 and 2017 than other year. Also, the seasonal for the seasonal fluctuation of the pollutants are not only caused by seasonal variation but also meteorological variable [19, 20].

### Seasonal meteorological variables

The variation of meteorological parameters was different in different seasons depending on the parameters. In general, the seasons in Thailand are classified into 3 seasons: the dry season or summer season starts from mid-February to mid-May, the rainy season occurs from mid-May to mid-October, and the winter season is the period from mid-October to mid-February. In this study, the analysis of differences among seasonal variation in measurable climatic parameters in both monitoring stations. The variation of climatic parameters was dissimilar in different seasons depending on the parameters. The difference was tested by ANOVA in each station as illustrated in Table 2. Concerning the climatic parameters, there was no difference in pressure in both stations for the rainy and winter seasons. A difference in temperature at station 65 between the rainy and winter seasons. Other climatic parameters are seasonal differences in both stations.

**Table 2:** Different analysis of seasonal variation for climatic parameters in each station

Station	Meteorological Factor	Contrast	*P*-value
Station 65	Temp	Rainy vs Winter	< 0.0001
		Rainy vs Summer	0.0230
		Summer vs Winter	< 0.0001
	Relative Humidity	Rainy vs Winter	< 0.0001
		Rainy vs Summer	< 0.0001
		Summer vs Winter	< 0.0001
	Pressure	Rainy vs Winter	0.0244
		Rainy vs Summer	< 0.0001
		Summer vs Winter	< 0.0001
Station 73	Temp	Rainy vs Winter	< 0.0001
		Rainy vs Summer	< 0.0001
		Summer vs Winter	< 0.0001
	Relative Humidity	Rainy vs Winter	< 0.0001
		Rainy vs Summer	< 0.0001
		Summer vs Winter	< 0.0001
	Pressure	Rainy vs Winter	0.2585
		Rainy vs Summer	< 0.0001
		Summer vs Winter	< 0.0001

The variation in PM_10_ concentrations based on Bonferroni multiple comparison test among different seasons is shown in Table 3. However, high PM_10_ concentration was observed in the summer period in both stations. Therefore, the mean comparison of PM_10_ concentration between seasons was carried out by using the Bonferroni method. According to the study, the mean concentration of PM_10_ was significantly higher during the summer than during the winter and rainy seasons combined in a year. The highest concentration was observed in summer, in both stations. The comparison of average PM_10_ concentration by season was determined by Bonferroni analysis is vary with shifting seasons [21]. Same as a study from Cichowicz et al. mention that seasonal variation of air pollution is associated with variety of seasons [22]. We found a significant difference in both stations as illustrated in Table 3 (*p* <  0.001).

**Table 3:** shows the seasonal variation of PM_10_ concentration by Bonferroni analysis

Pollution	Station	Contrast	*P*-value
PM_10_	Station 65	Rainy vs Winter	< 0.0001
		Rainy vs Summer	< 0.0001
		Summer vs Winter	< 0.0001
	Station 73	Rainy vs Winter	< 0.0001
		Rainy vs Summer	< 0.0001
		Summer vs Winter	< 0.0001

### Comparison of MLR models

The MLR results are obtained using the annual data of Chiang Rai province. Even though available data related to PM_10_ has indicated different seasons, they have been fitted for each season to examine their respective regression presentations. The coefficients corresponding to the different seasonal models are shown in Table 4. From the obtained models, it can be explained that CO was the dominated parameter of PM_10_ concentration. For example, in the annual model of both stations, the coefficient of CO was 56.6 in station 65, compared to 1.3 of temperature, 0.3 of humidity, and 0.7 of pressure. It indicated that the change of CO 1 unit induced the change of PM_10_ concentration of 56.6 μg/m^3^.

**Table 4:** Comparison of multiple linear regression in two different monitoring sites

Chiang Rai (Station 65)	Chiang Rai (Station 73)
*Y*_*a*_ = 46.9 + 56.6*x*_1_ + 1.3*x*_2_ + 0.3*x*_3_ + 0.7*x*_4_	*Y*_*a*_ = 22.1 + 45.2*x*_1_ + 1.1*x*_2_ + 0.4*x*_4_
*Y*_*s*_ = 1486.0 + 116.8*x*_1_ + 0.7*x*_2_ + 0.6*x*_3_ − 0.9*x*_4_ − 1.5*x*_5_	*Y*_*s*_ = − 87.3 + 54.4*x*_1_ + 1.5*x*_2_ + 2.6*x*_3_
*Y*_*r*_ = 67.0 + 9.3*x*_1_ + 0.4*x*_2_ + 0.03*x*_3_ − 0.3*x*_4_ − 0.03*x*_5_	*Y*_*r*_ = − 124.0 − 6.11*x*_1_ + 4.4*x*_3_ + 0.5*x*_4_
*Y*_*w*_ = 11.4 + 10.7*x*_1_ + 1.5*x*_2_ + 0.7*x*_3_ − 0.2*x*_4_ + 0.03*x*_5_	*Y*_*w*_ = − 33.9 + 58.4*x*_1_ + 1.9*x*_2_

Here, *Y* = *The concentration of PM*_10_ (a =*annual*, s = *summer*, r = *rainy*, *and* w = *winter*), *x*_1_ = *CO*, *x*_2_ = *O*_3_, *x*_3_ = *Temperature*, *x*_4_ = *Relative humidity and x*_5_ = *Air Pressure*

**Fig. 3:**
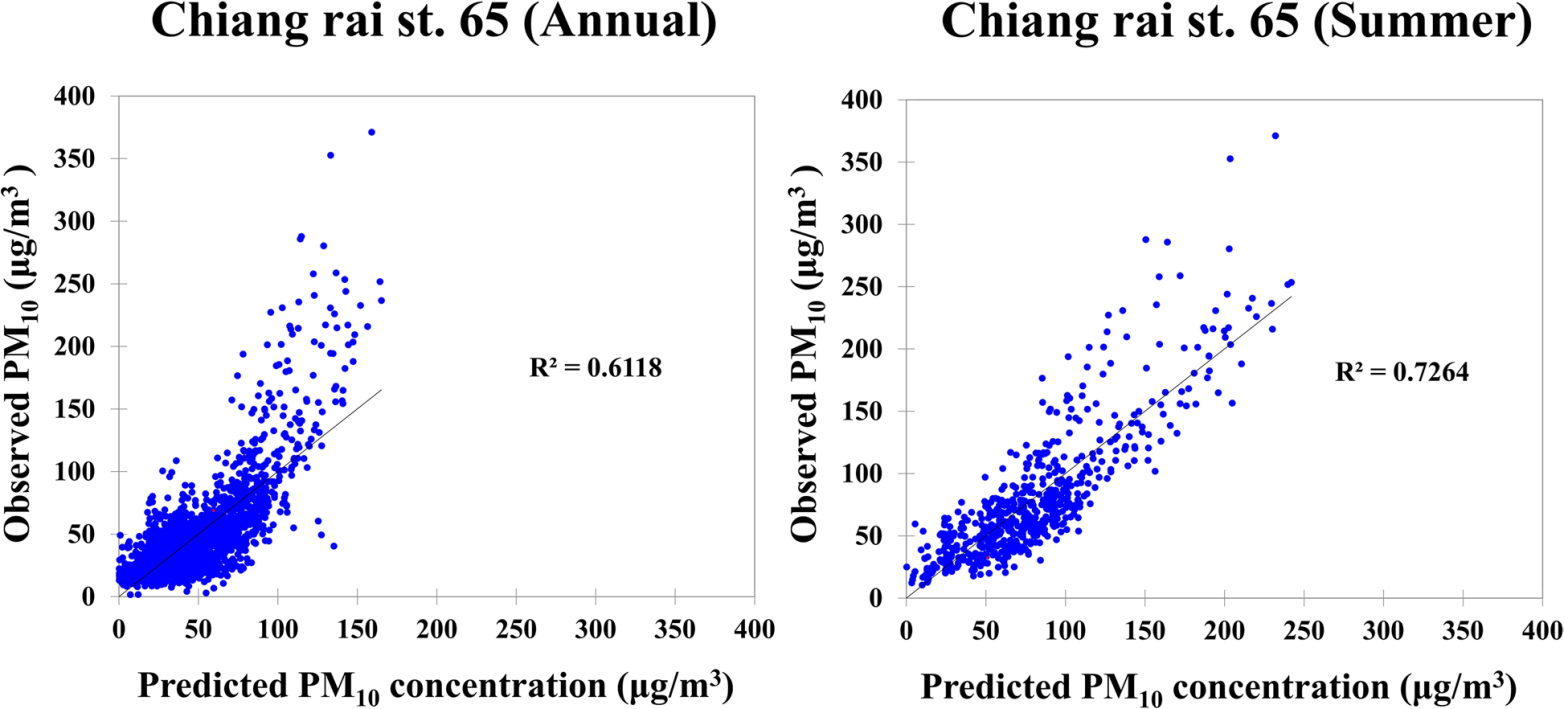
Fitting results of PM_10_ data of Ching rai (station 65) in annually and summer season

**Fig. 4:**
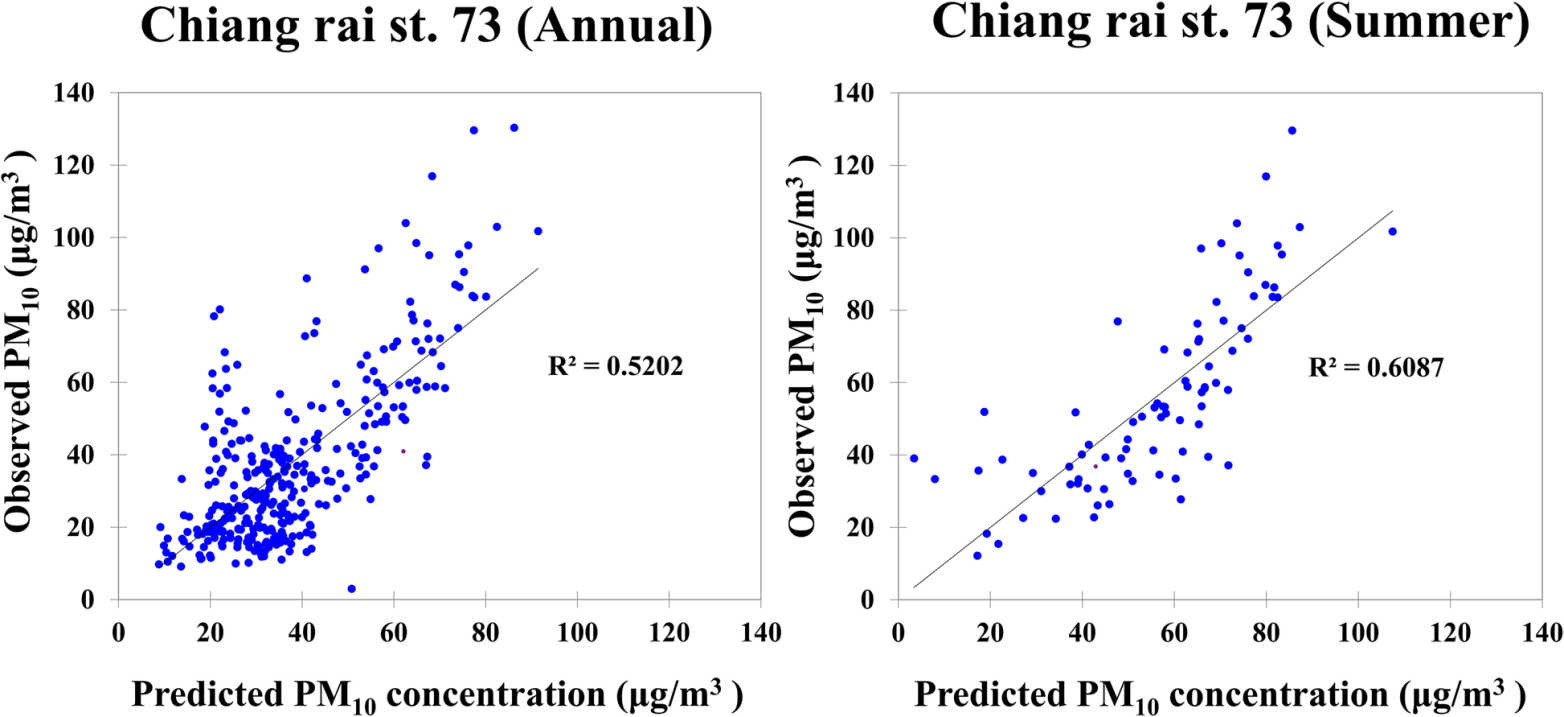
Fitting results of PM_10_ data of Ching rai (station 73) in annually and summer season

Figures 3 and 4 shows the scatter plot for the model fitting of Chiang Rai’s PM_10_ data from 2011 to 2018. The fitted line was generated by Excel software packaging, which is based upon the least squares method to find out the linear trend with the best fitness among the scattered points. R^2^ and RSME for the MLR model in annual data from station 65 (Fig.3) were 0.61 and 22.15 μg/m^3^, respectively. In the summer, it was 0.73 and 27.95 μg/m^3^ respectively. In station 73 (Fig. 4), R^2^ and REME were 0.52 and 15.83 μg/m^3^ annually, 0.61 and 16.45 μg/m^3^ for summer respectively, and the range of VIF for the independent variable was lower than 10 as 1.07–2.47 [12], which indicated that there was no multi-collinearity in variables. Moreover, the Durbin-Watson test showed that the range values for all models were still within the 0–4 range; Station 65 was 0.63, 0.41, 0.85 and 0.64 for PM_10_ annually PM_10_, summer, PM_10_, rainy, and PM_10_, winter respectively, and for station 73 were 0.67, 0.98, 0.64 and 0.1.18 for PM_10_, annual, PM_10_, summer, PM_10_, rainy, and PM_10_, winter respectively. Thus, it indicates that all of the models do not have any first-order autocorrelation problems as the range values [12].

Chiang Rai is a tropical zone and has a temperate monsoon climate characterized by precipitous, hot summers and other specific seasonal characteristics. The PM_10_ monitoring data were further classified into three seasons: summer (mid-February to mid-May); rainy (mid-May to mid-October); and mid-October to mid-February. As can be seen in Table 4, the mean PM_10_ concentrations for summer and winter exceeded those of the rainy season. Table 5 shows that the results of PM_10_ regression in the three seasons show high fitness for summer and winter, both with R^2^ greater than 0.40; however, the rainy season is lowest, with a R^2^ of only 0.12–0.24.

**Table 5:** Summary of R^2^ and error measure for fitting model

Seasonal Regression	Fitting results			
	***R***^**2**^	RMSE	VIF	DW
**Station 65**				
Annual	0.61	22.15	1.07–1.59	0.63
Summer	0.73	27.95	1.19–1.39	0.41
Rainy	0.24	8.88	1.14–1.57	0.58
Winter	0.40	13.40	1.01–1.27	0.64
**Station 73**				
Annual	0.52	15.83	1.01–1.68	0.67
Summer	0.61	16.45	1.03–1.17	0.98
Rainy	0.12	13.72	1.10–2.47	0.64
Winter	0.67	11.06	1.00	1.18

The correlation between PM_10_ and the other parameters and variables is shown in Table 6, During the study period, there was an extremely strong correlation between the mean concentration of PM_10_ in the summer season and those of CO (*r* = 0.7, 0.5), and O_3_ (*r* = 0.5, 0.6). In Chiang Rai province, PM_10_ concentrations were negatively correlated with RH (*r* = − 0.6, − 0.6) in all seasons, suggesting that the high humidity level allows PM_10_ removal. Sometimes the increment in rainfall occurrence is accompanied by in-cloud scavenging [6], and relative humidity influences particle movement and can settle PM_10_ at ground level [20]. On the other hand, the correlations with temperature were strongly positive in all seasons except for the winter, which is due to the significant role temperature plays in particulate matter. According to the high PM_10_ concentrations during warm days, which can be related to enhanced photochemical activity on days with high solar intensity and the possible formation of secondary particulate matter [6, 23].

**Table 6:** Summary of seasonal by Spearman’s rank correlation coefficient (*r*) coefficient

Parameter	Station 65						Station 73					
Summer	PM_10_	CO	O_3_	Temp	RH	P	PM_10_	CO	O_3_	Temp	RH	P
PM_10_	**1.00**	**0.71**	**0.47**	**0.31**	**−0.60**	**−0.17**	**1.00**	**0.46**	**0.56**	**0.26**	**− 0.58**	**− 0.42**
CO	**0.71**	1.00	0.20	0.05	−0.34	0.07	**0.46**	1.00	0.24	0.21	−0.53	− 0.02
O_3_	**0.47**	0.20	1.00	0.39	−0.38	−0.23	**0.56**	0.24	1.00	0.38	−0.35	−0.43
Temp	**0.31**	0.05	0.39	1.00	−0.35	−0.27	**0.26**	0.21	0.38	1.00	−0.40	−0.63
RH	**−0.60**	−0.34	− 0.38	−0.35	1.00	0.19	**−0.58**	−0.53	− 0.35	−0.40	1.00	0.25
P	**−0.17**	0.07	−0.23	−0.27	0.19	1.00	**−0.42**	−0.02	− 0.43	−0.63	0.25	1.00
Rainy	PM_10_	CO	O_3_	Temp	RH	P	PM_10_	CO	O_3_	Temp	RH	P
PM_10_	**1.00**	**0.29**	**0.30**	**0.36**	**−0.42**	**0.15**	**1.00**	**−0.10**	**−0.10**	**0.25**	**−0.23**	**− 0.31**
CO	**0.29**	1.00	−0.06	0.08	0.06	0.06	−0.10	**1.00**	−0.41	0.33	−0.02	−0.72
O_3_	**0.30**	−0.06	1.00	0.40	−0.38	0.07	−0.10	**−0.41**	1.00	−0.27	− 0.32	0.59
Temp	**0.36**	0.08	0.40	1.00	−0.61	0.08	0.25	**0.33**	−0.27	1.00	−0.80	−0.64
RH	**−0.42**	0.06	−0.38	−0.61	1.00	−0.16	− 0.23	**−0.02**	− 0.32	−0.80	1.00	−0.14
P	**0.15**	0.06	0.07	0.08	−0.16	1.00	−0.31	**−0.72**	0.59	−0.64	− 0.14	1.00
Winter	PM_10_	CO	O_3_	Temp	RH	P	PM_10_	CO	O_3_	Temp	RH	P
PM_10_	**1.00**	**0.28**	**0.60**	**−0.26**	**−0.41**	**0.27**	**1.00**	**0.00**	**0.59**	**−0.34**	**−0.24**	**0.29**
CO	**0.28**	1.00	0.09	−0.01	−0.07	0.09	**0.00**	1.00	−0.06	0.09	−0.27	0.26
O_3_	**0.60**	0.09	1.00	−0.20	− 0.46	0.10	**0.59**	−0.06	1.00	−0.23	− 0.71	0.21
Temp	**−0.26**	−0.01	− 0.20	1.00	0.08	−0.20	**− 0.34**	0.09	− 0.23	1.00	0.07	−0.79
RH	**−0.41**	−0.07	− 0.46	0.08	1.00	−0.16	**− 0.24**	−0.27	− 0.71	0.07	1.00	−0.07
P	**0.27**	0.09	0.10	−0.20	−0.16	1.00	**0.29**	0.26	0.21	−0.79	−0.07	1.00

### PM_10_ dispersion and backward air mass trajectory analysis

The peak of PM_10_ concentration (Fig. 2), recorded at Chiang Rai station, was found in March of 2012 to 2016, and April of 2011 and 2018. The weather data was obtained from the National Oceanic and Atmospheric Administration (NOAA) website by identifying the locations of both sites. The trajectory map indicated that 13 days of air movement were generated from neighboring countries from 24 days of records in Chiang Rai station (supplement 1). While at Mae Sai District Station (station 73), we discovered 20 days of air moved to a neighboring country [17, 18]. However, the weather in Mae Sai district is likely to be affected partially by the PM_10_ invented in neighboring countries. More than Chiang Rai Station (station 65).

## Conclusion

The PM_10_ concentration levels and meteorological data of Chiang Rai province were collected from 1 January 2011 to 31 December 2018 (Station 65) and 1 July 2011 to 31 December 2018 (Station 73). The higher levels of PM_10_ were observed in Chiang Rai province (station 73) with values ranging from 3.0 μg/m^3^ to 479.1 μg/m^3^ and a mean concentration of 52.3 μg/m^3^. Temperature relative to humidity and pressure provide the highest influence on the level of PM_10_ concentration. Relative humidity and pressure showed an inverse relationship, thus a decrease in PM_10_ impact, even though temperature showed a positive association with PM_10_ concentrations. The difference in PM_10_ concentration between dry and wet seasons can be caused by scavenging processes in rain in the wet seasons. According to the MLR model, the influences of CO, O_3_, RH, temperature, and pressure on PM_10_ concentrations during the annual, summer, and winter seasons are significant. The R^2^ values for the annual summer, rainy, and winter seasons are 0.61, 0.73, and 0.40 (station 65) and 0.52, 0.61, and 0.67 (station 73), respectively. This research concerned only temperature, relative humidity, pressure, and other meteorological factors to determine the relationships, but the effects of other parameters are well documented and, thus, future studies will have more added variables to solve the issue more efficiently.

## Supplementary Information

**Additional file 1 **: **Supplement 1.** Show the backwards trajectory of AQMS in Chiang rai province.

## Data Availability

Datasets used and/or analyzed during this study are available from the corresponding author upon reasonable request.
