# Supplementary figures and images for: Particulate matter (PM10) prediction based on multiple linear regression: a case study in Chiang Rai Province, Thailand
Source: BMC Public Health. 2021 Nov 24;21:2149. doi: 10.1186/s12889-021-12217-2 (PMC8611941; doi:10.1186/s12889-021-12217-2)

**Supplement 1.** Show the backwards trajectory of AQMS in Chiang rai province.

| 2011 | 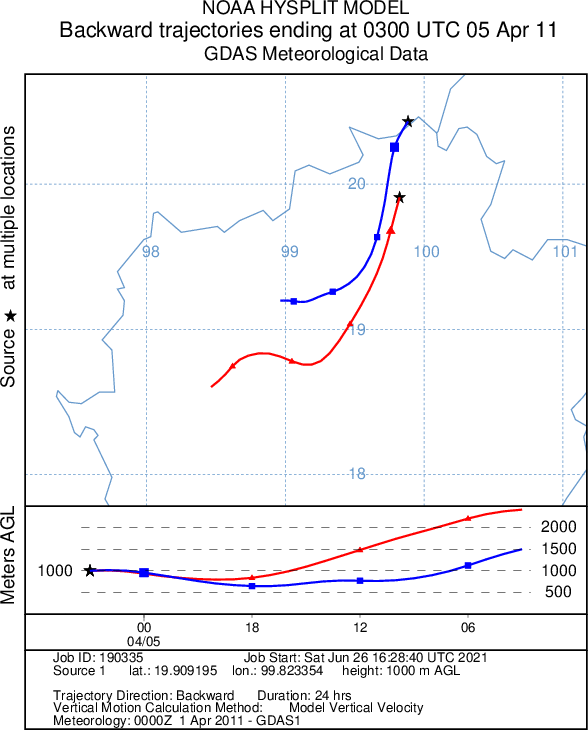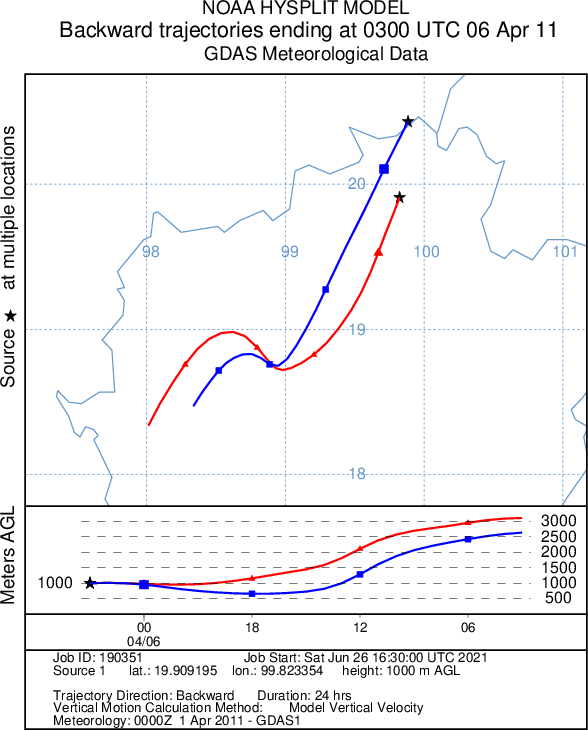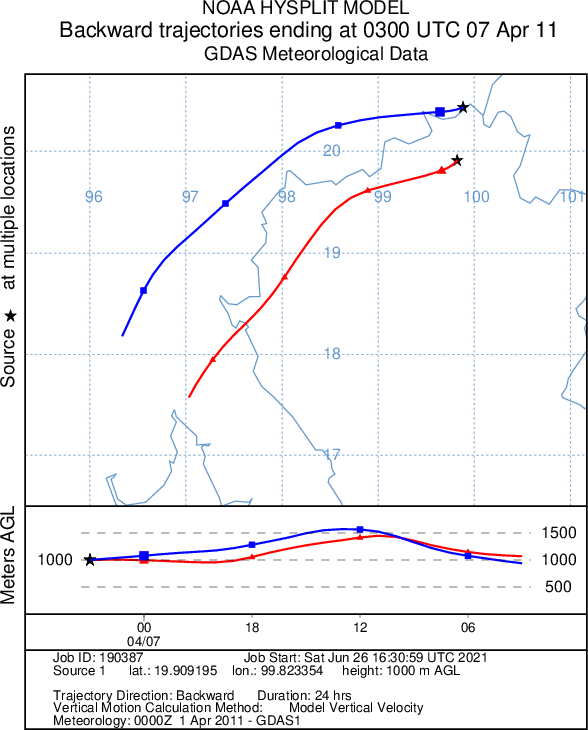 |
| --- | --- |
| 2012 | 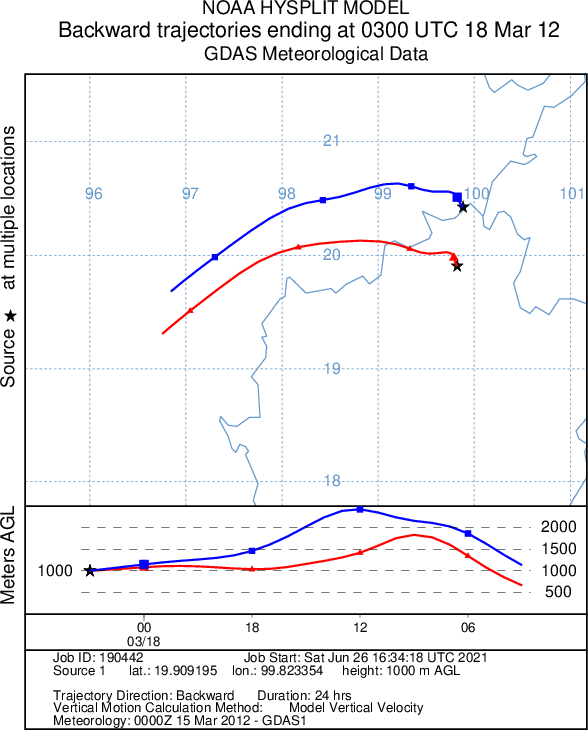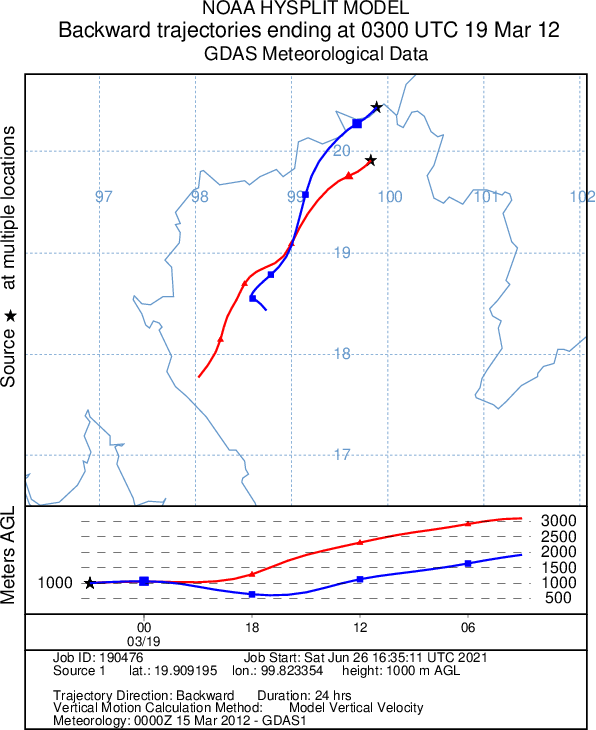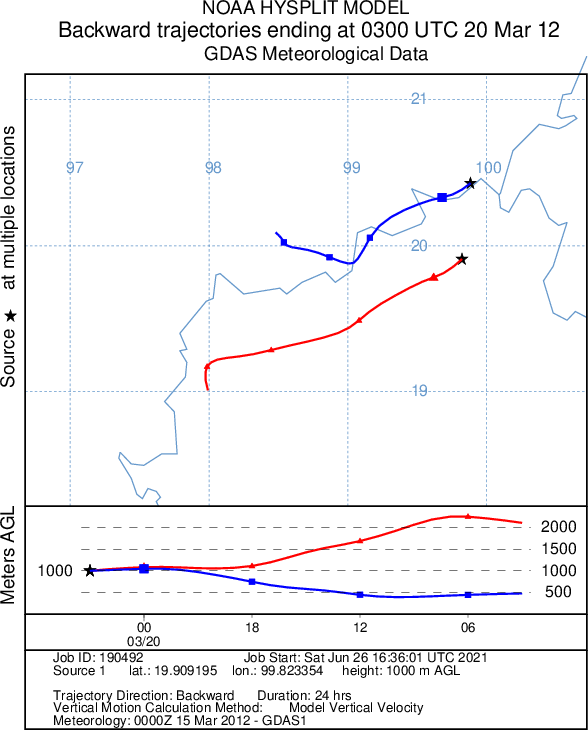 |
| 2013 | 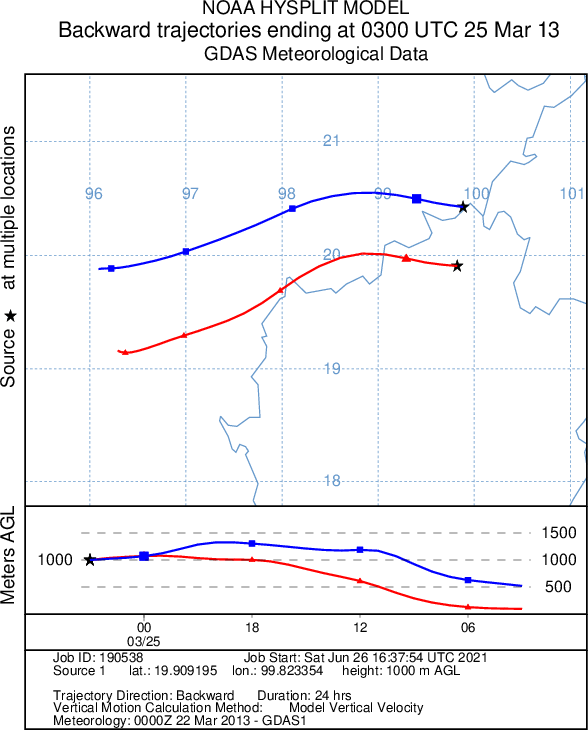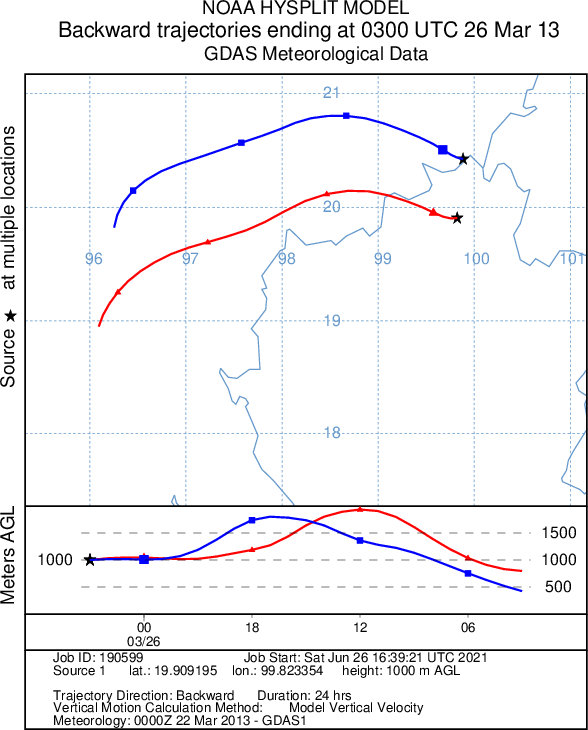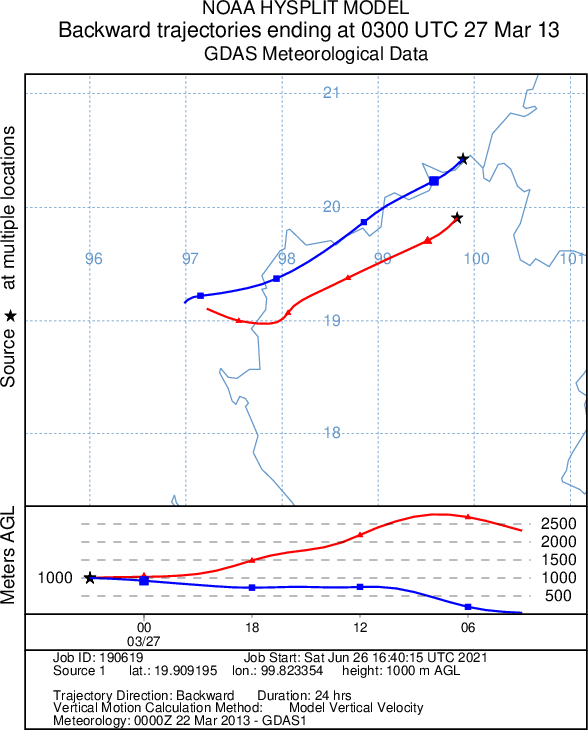 |
| 2014 | 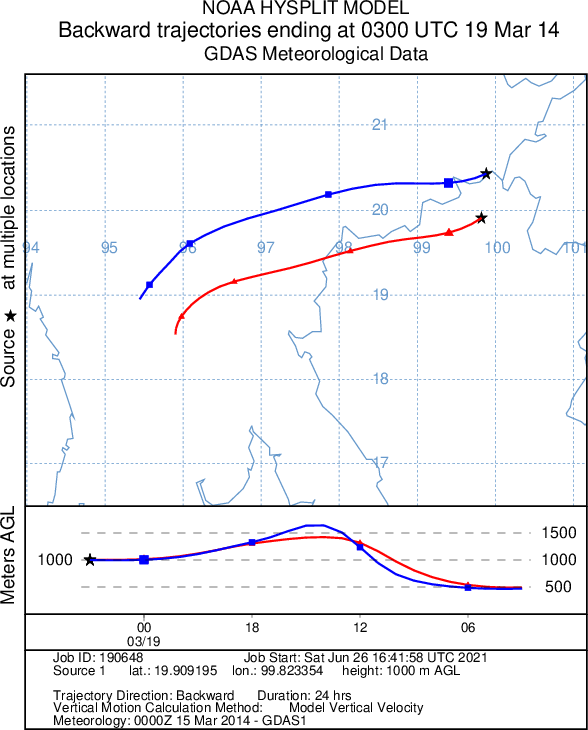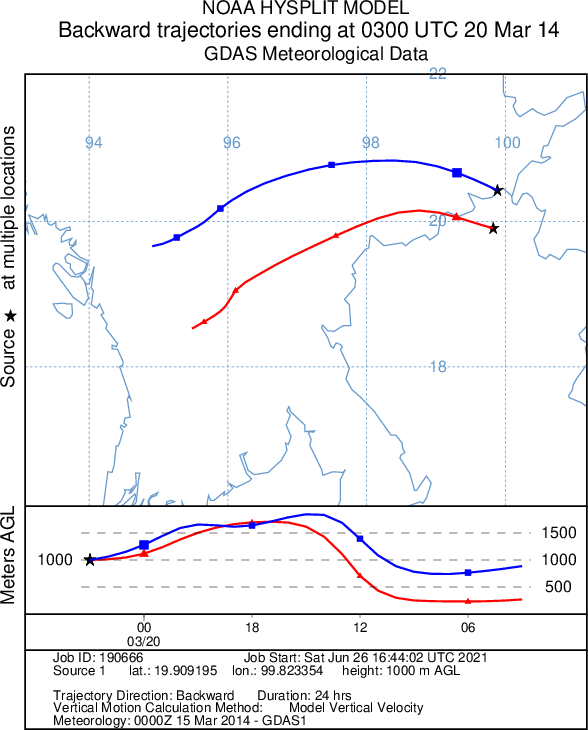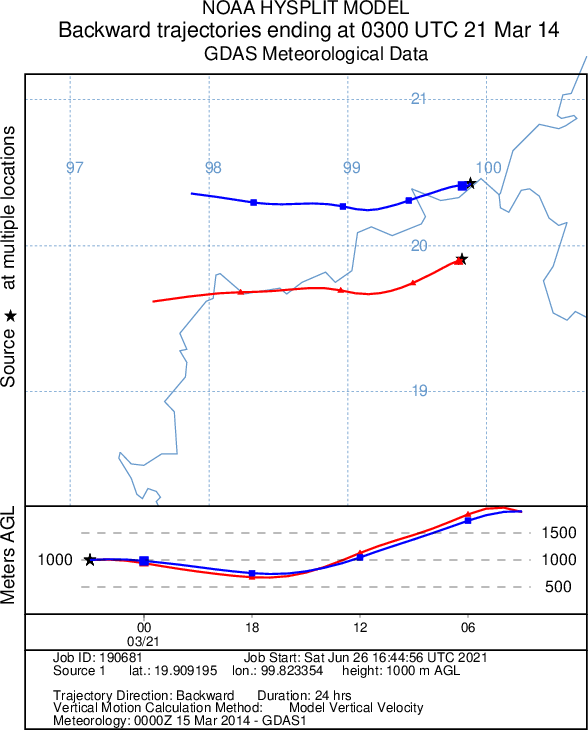 |
| 2015 | 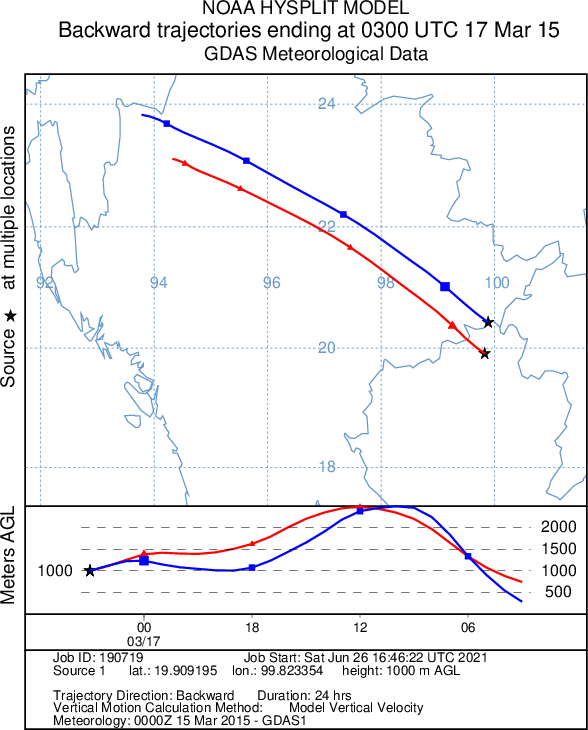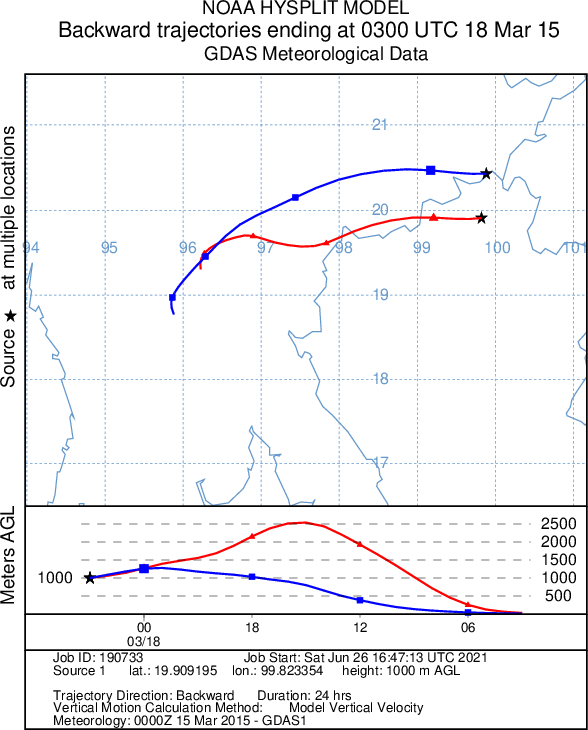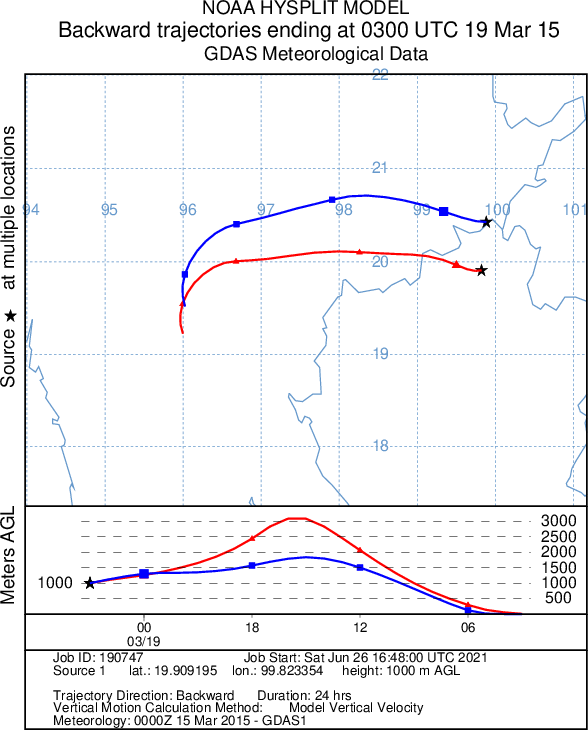 |
| 2016 | 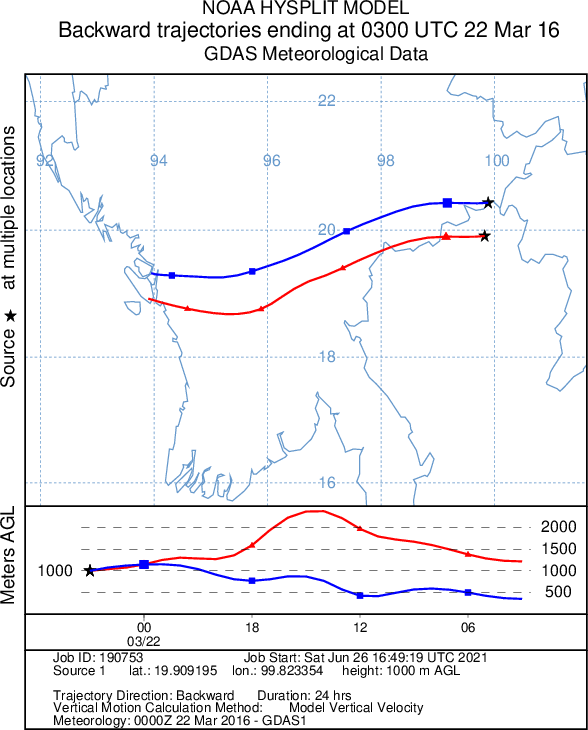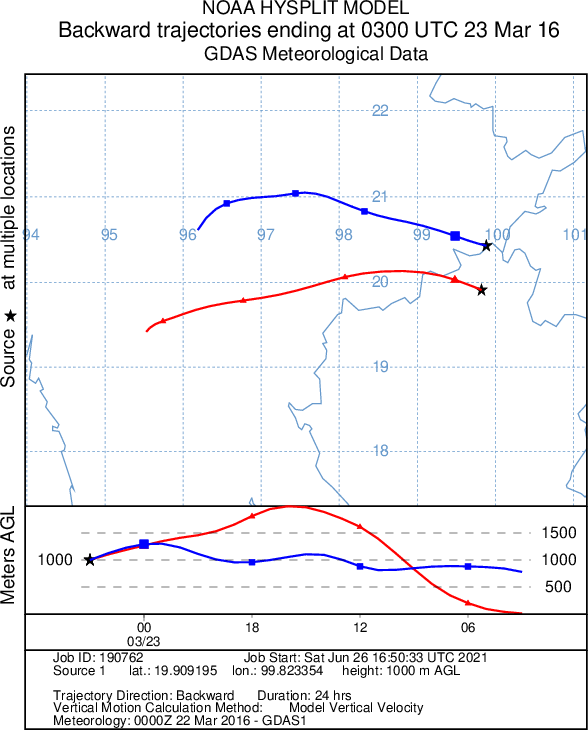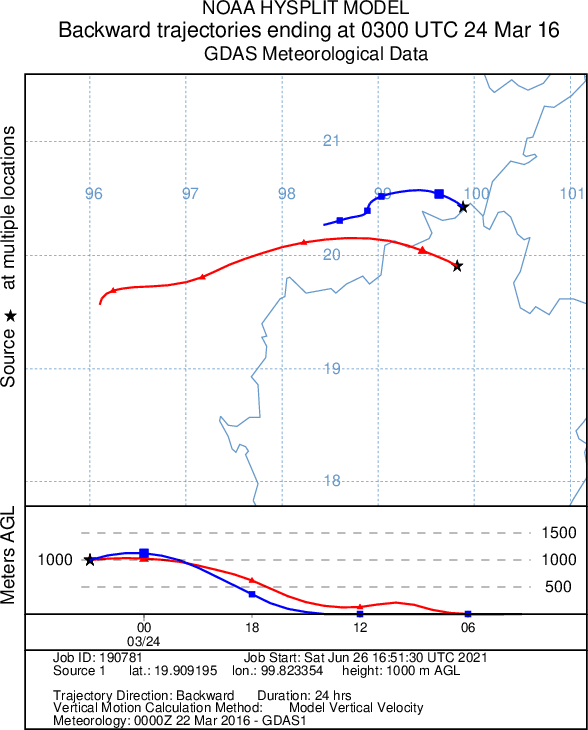 |
| 2017 | 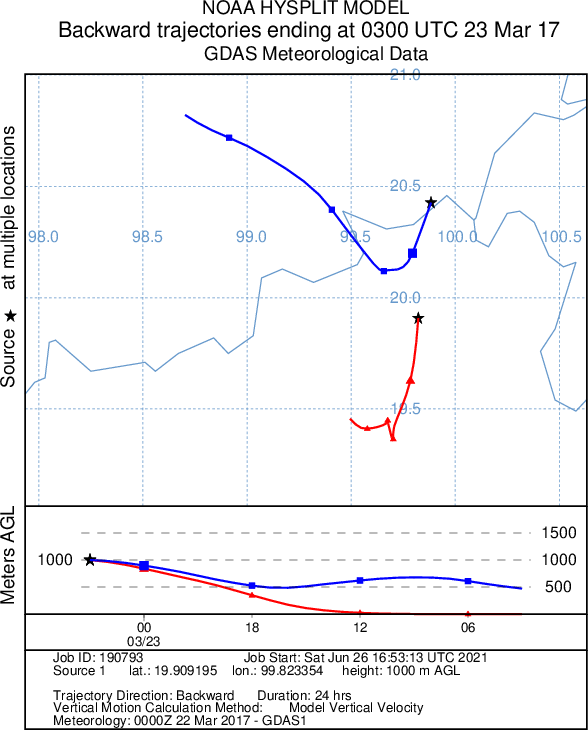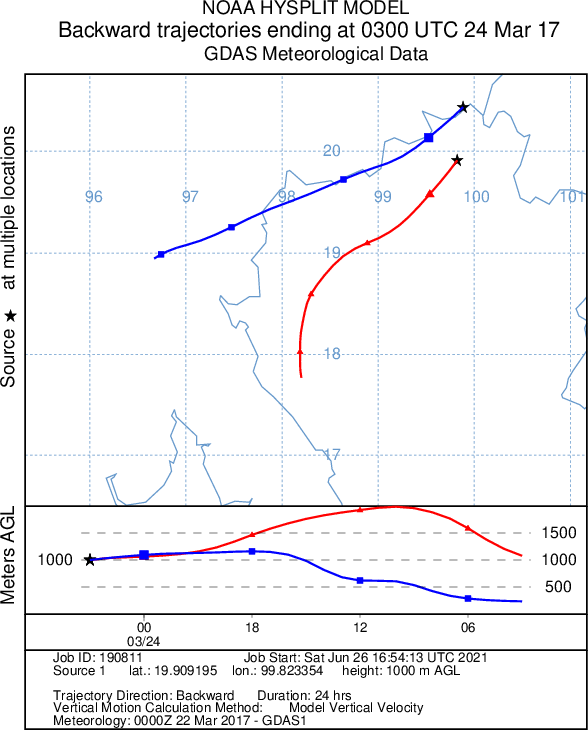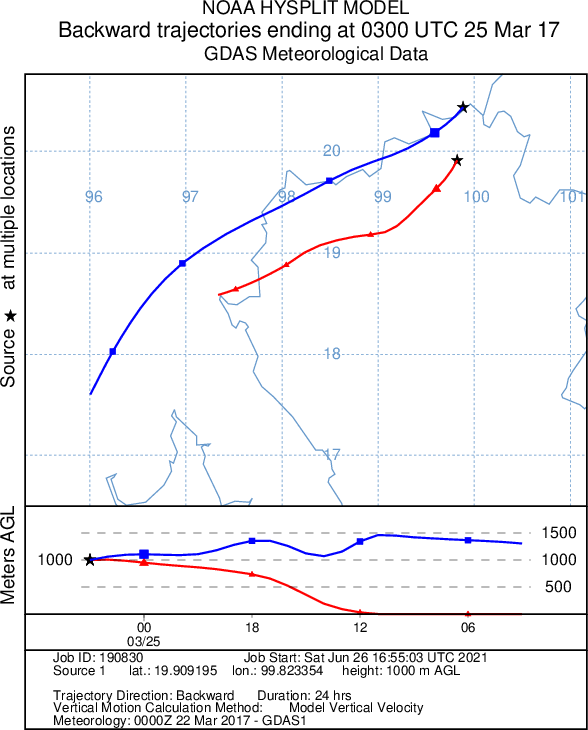 |
| 2018 | 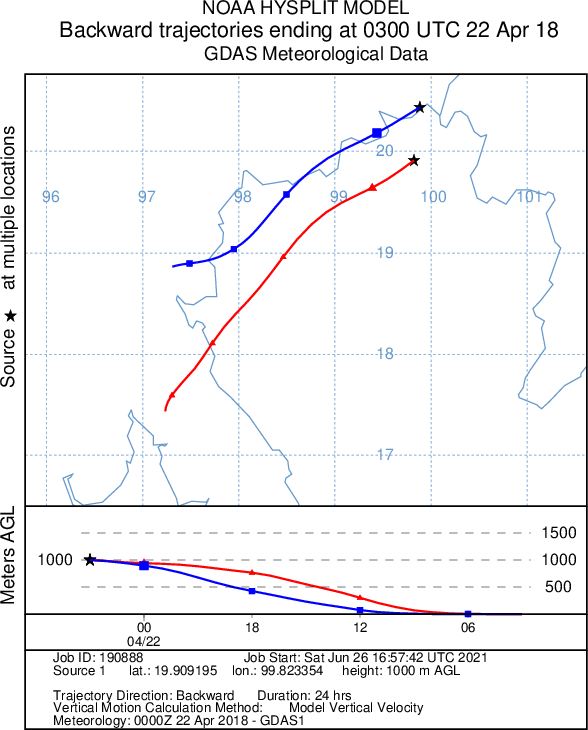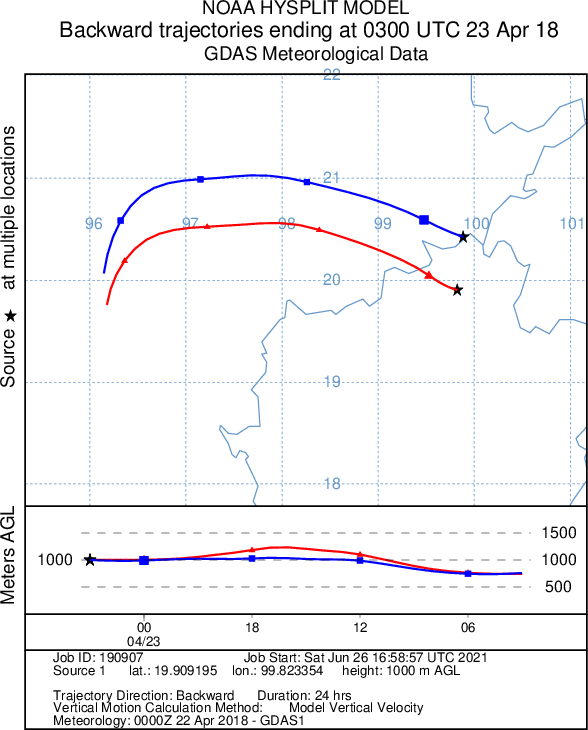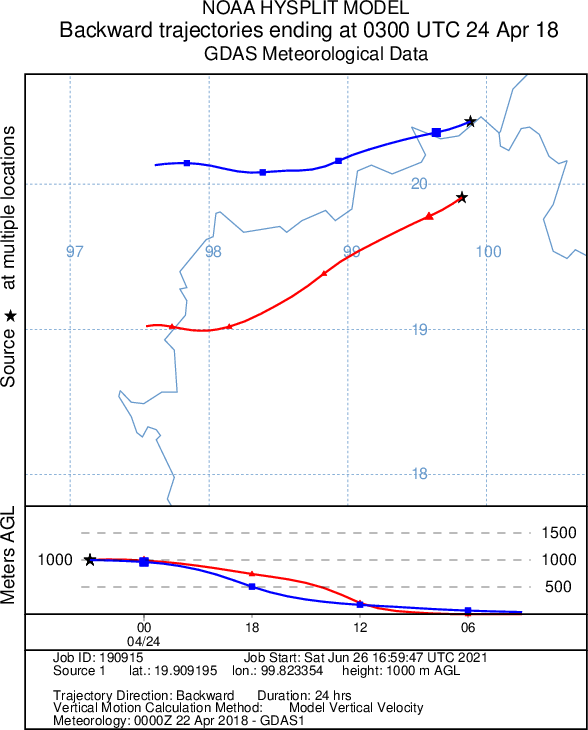 |

Supplement: Supplementary file 1 — Additional file 1 : Supplement 1. Show the backwards trajectory of AQMS in Chiang rai province. [file 12889_2021_12217_MOESM1_ESM.docx]
